# Cardiorespiratory Fitness Is Associated with a Reduced Cardiovascular Risk in Occupational Groups with Different Working Conditions: A Cross-Sectional Study among Police Officers and Office Workers

**DOI:** 10.3390/jcm10092025

**Published:** 2021-05-09

**Authors:** Markus Strauss, Peter Foshag, Anna Brzęk, Richard Vollenberg, Ulrich Jehn, Henning Littwitz, Roman Leischik

**Affiliations:** 1 Department of Cardiology, Faculty of Health, School of Medicine, University Witten/Herdecke, 58095 Hagen, Germany; peter.foshag@uni-wh.de (P.F.); h.littwitz@gmx.de (H.L.); 2 Department of Cardiology I-Coronary and Peripheral Vascular Disease, Heart Failure Medicine, University Hospital Muenster, 48149 Muenster, Germany; 3 Department of Physiotherapy, School of Health Sciences, Medical University of Silesia, 40000 Katowice, Poland; aniabrzek@interia.pl; 4 Department of Medicine B, Gastroenterology and Hepatology, University Hospital Muenster, 48149 Muenster, Germany; richard.vollenberg@ukmuenster.de; 5 Department of Medicine D, Division of General Internal Medicine, Nephrology and Rheumatology, University Hospital of Muenster, 48149 Muenster, Germany; ulrich.jehn@ukmuenster.de

**Keywords:** police officer, office worker, cardiovascular prevention, cardiorespiratory fitness, risk profile, cardiovascular risk factors, cardiovascular risk

## Abstract

Several studies reported a high prevalence of cardiovascular risk factors among police officers and office workers, and adequate cardiorespiratory fitness was reported to have protective effects in reducing cardiovascular risk. Therefore, the present study aimed to evaluate the effects of cardiorespiratory fitness on reducing cardiovascular risk factors in these occupational groups. This cross-sectional study enrolled 101 male participants (55 police officers and 46 office workers). Cardiorespiratory fitness was assessed via spiroergometry. Cardiovascular risk factors were also examined, and the 10-year cardiovascular risk and heart/vascular age were reported using the Framingham risk score. In both groups, higher cardiorespiratory fitness was associated with lower cardiovascular risk factors. Police officers and office workers with higher cardiorespiratory fitness demonstrated significantly lower values in BMI, waist circumference, body fat percentage, diastolic resting blood pressure, heart rate, triglycerides and total cholesterol values, and 10-year cardiovascular risk and heart/vascular age (all factors *p* < 0.0077, age adjusted). Police officers and office workers mostly presented low levels of cardiorespiratory fitness: 60% of police officers and 58% of office workers were considered “not fit and obese”. Despite different working conditions, both occupational groups had a high rate of low cardiorespiratory fitness levels and showed no differences in their cardiovascular risk profiles. In both groups, cardiorespiratory fitness reduced cardiovascular risk factors, but there was no difference in the influence of cardiorespiratory fitness on cardiovascular risk factors.

## 1. Introduction

One of the most important factors for protection against cardiovascular diseases is physical activity [1,2]. Previous studies showed that professional work with high physical activity protects against cardiovascular disease [3]. On the other hand, Coenen et al. [4] demonstrated that men with a high level of occupational physical activity had an 18% increased risk of early mortality compared to those engaged in low levels of occupational physical activity. Overall, there are great differences in physical activity levels between different professions. Today, physical activity and health plasticity are additionally dependent on environmental circumstances [5]. Moreover, possible pandemics (e.g., COVID-19), rapid globalization, urbanization, an aging society, and an increase in chronic diseases pose new and complex challenges to all health care systems [6,7]. A sedentary work profession may lead to higher mortality if the worker’s lifestyle behaviors during leisure time influence mortality in a negative manner. This issue is multifaceted and is also strongly affected by income. The German police officers in our study had a moderate-income, generally sedentary work characteristics, and low counter-regulation.

The working conditions of office worker are characterized by predominantly sedentary work [8]. A meta-analysis by van Uffelen et al. [9] showed negative associations between sitting time at work and increased cardiometabolic biomarkers and risk. Studies from different countries have frequently reported unhealthy lifestyles and physical inactivity among office workers [10]. In contrast to office workers, working as a police officer is known to be dangerous. Thus, to ensure that the police officer remains safe, good physical fitness is mandatory. Police officers’ working conditions, however, are inhomogeneous and differ depending on the officer’s main work activity. Some police officers mainly carry out sedentary office activities, whereas others primarily engage in physically demanding work during their everyday patrol services. Past studies have indicated a high prevalence of risk factors for cardiovascular disease among police officers [11]. Exposure to physical stress among police officers was associated with obesity, dyslipidemia, and hypertension as well as impaired glucose metabolism [12]. However, physical fitness among police officers has not yet been systematically reviewed in the literature. A current systematic review of police officers’ physical fitness levels provided values similar to or above the average among the general population [13]. On the other hand, subsequent studies provide growing evidence that the physical fitness of police officers is below the recommended standards for general health [14].

Police officers require good fitness for emergency situations, as do all who work in the area of public safety (e.g., firefighters and medical staff) [15,16]. Adequate physical fitness is especially necessary on active patrol duty. Izawa et al. [17] noted that even police officers in the administrative service must always expect to be deployed on patrol duty.

The physical demands of on-duty policemen can be high at any given time. For example, a police officer may be at rest but then suddenly have to intervene in an emergency situation. However, accelerometer measurements suggest that male police officers engage in less job-related physical activity than secretaries and appear to be more active on their off-duty days than during their work hours [18]. However, if physical activity is not pursued during off-duty periods, police officers tend to become obese. Additionally, there is a correlation between activity levels and areas of police duty, which can require different demands. In general, police officers who are more stressed tend to be less active [18,19].

Under this background, the present study investigates whether higher cardiorespiratory fitness (CRF) is associated with cardiovascular risk factor (CVRF)reductions for police officers and office workers, who represent two groups with different working conditions.

## 2. Materials and Methods

### 2.1. Study Population

This cross-sectional study included 101 male participants (55 police officers and 46 office workers) from North-Rhine-Westphalia, Germany. The inclusion criterion for police officers was employment in shift-based police-patrol work with the federal police force. Office workers were employed in administrative offices involving predominantly sedentary tasks. These workers were employed in tax offices or municipal administration and performed desk work. Desk work was characterized as sedentary work in a full-time job (>35 h per week) in accordance with the criteria of the Sedentary Behavior Research Network (SBRN) [20]. Examinations were performed at the Center of Sports Medicine in Hagen by a trained clinical physician.

The ethical review board of the University Witten/Herdecke approved the study, which was performed in accordance with the ethical principles in the Deceleration of Helsinki. Written informed consent was obtained from all participants.

### 2.2. Assessment of Cardiovascular Risk Factors (CVRF)

On day one of the examination, the anthropometric characteristics, participant histories, resting heart rate and blood pressure measurements, and blood samples were obtained.

Using a questionnaire, each individual’s nicotine consumption (cig./day) and years of professional experience were determined. Body weight, body fat (%), and body composition were measured using a Tanita BC-418MA Segmental Body Composition Analyzer (Tanita Corporation, Tokyo, Japan). The measurement of height in a standing position was performed using a clinic stadiometer. Body mass index (BMI) was calculated as the weight in kilograms divided by the square of height in meters. In a standing position, waist circumference was measured at the center of the lower edge of the ribs and the iliac crest’s upper edge.

Diastolic (RDBP) and systolic (RSBP) resting blood pressure and heart rate (HR) were measured after a five-minute break in the supine position. In this position, blood pressure was measured three times at intervals of one minute. The mean value of these three bloodpressure measurements was taken as the resting blood pressure. HR measurements were performed using a 12-lead ECG.

To ensure valid blood collection, participants were asked to fast (no eating and drinking for 6 h prior to examination). Venous blood collection included the following parameters: total cholesterol, high-density lipoprotein (HDL), low-density lipoprotein (LDL), and triglycerides.

Calculation of the cardiovascular risk profile and heart/vascular age was based on the Framingham score [21]. This study used the 10-year cardiovascular risk score calculator and heart/vascular age calculator [22]. The following parameters were considered in the calculations: age, the presence of diabetes mellitus, smoking, the presence of treated or untreated systolic blood pressure, total cholesterol, HDL levels, and BMI. The 10-year cardiovascular risk was divided into three risk groups: <10% low risk, 10–20% moderate risk, and >20% high risk.

### 2.3. Assessment of Cardiorespiratory Fitness (CRF)

Exercise spiroergometry testing with an electrocardiogram and estimation of oxygen consumption (VO2max/METs) was performed to assess cardiorespiratory fitness [23]. Spiroergometry exercise testing was performed using a ramp protocol starting at 50 watts and increasing the power by 25 watts every 2 min. Criteria for the end of the test were a subjectively exhausted participant with no further increase in maximal oxygen uptake for 20 s, achieving 85% of the individual maximum predicted heart rate (defined as 220-age), and/or the proband being unable maintain a cycle pace of 80 rotations per minute. The metabolic equivalent (MET) values achieved during spiroergometry were measured and analyzed. One MET was considered 3.5 mL·kg^−1^·min^−1^, which is often characterized as the metabolic cost of resting quietly [24].

The participants were divided into four groups based on their achieved METs to describe their cardiorespiratory fitness. The four groups were as follows: very low (≤10 METs), low (10 to 12 METs), intermediate (>12 to 14 METs), and high fitness levels (>14 METs).

### 2.4. Statistical Analysis

Stata/IC 13.1 for Windows (StataCorp LP, College Station, TX, USA) was used for the statistical analysis. The baseline characteristics here are described using the mean, standard deviation (SD), and median. For the categorical variables, frequency is reported. Quantitative variables are presented based on an analysis of variance, and the x^2^ test was used for a group comparison of the categorical variables. To describe the influence of MET and both MET and BMI on CVD risk factors, linear regression models were determined. These descriptions were produced using different models (unadjusted, adjusted for age, adjusted for age, and BMI). The statistical tests were two-sided with a significance level of 0.05.

## 3. Results

### 3.1. Baseline Characteristics

The baseline characteristics of the participants are summarized in Table 1.

The examined police officers had an average age of 45.3 ± 7.8 years, whereas the office workers were, on average, 45.8 ± 10.0 years old. Based on the international BMI classification, 61.8% of the police officers and 43.5% of the office workers were overweight and 23.6% of the police officers and 13.0% of office workers were obese. Normal weight was measured for 14.5% of police officers and 43.5% of office workers. The average waist circumference of the police officers was 97.8 ± 12.4 cm, and that of office workers was 97.3 ± 11.7 cm. The average values of both groups were above the reference value of 84 cm according to the International Diabetes Foundation. The MET score was used for measuring CRF in both groups. Police officers and office workers achieved the same average METs of 9.7 ± 2.3. In most cases, both groups achieved METs ≤10 (police officers: 54.5%; office workers: 58.7%). METs scores of > 10 to ≤ 12 were obtained by 23.9% of police officers and 27.3% of office workers, and METs scores of 12 to ≤ 14 were achieved by 17.4% of police officers and 16.4% of office workers. Only one police officer (1.8%) and no office workers achieved METs > 14. Police officers presented an average 10-year cardiovascular risk of 9.6 ± 7.4% and a heart/vascular age of 51.1 ± 13.9 years. The 10-year cardiovascular risk of office workers (9.7 ± 9.2%) was nearly the same as that of police officers, but the average heart/vascular age of office workers was lower than that of police officers (49.6 ± 15.5 years).

### 3.2. Association between Cardiovascular Risk Factors and Cardiorespiratory Fitness

Table 2 summarizes the associations of CVRF and CRF among the police officers and office workers. These results are presented using three different analytical models: unadjusted, adjusted for age, and adjusted for both age and BMI. Only one police officer and no office workers were included in the METs >14 group. Consequently, no valid observations could be derived, and the METs > 14 group is not reported in Table 2. The unadjusted analysis indicates significant associations between higher CRF and lower BMI, lower waist circumference, lower body fat, lower HR, lower triglycerides (only police officers), lower total cholesterol, lower HDL-cholesterol, lower LDL-cholesterol, lower professional experience (only police officers), and lower 10-year cardiovascular risk and heart/vascular age in both groups (police officers and office workers) (all factors *p* < 0.0483).

When adjusted for age, significantly higher values of BMI, waist circumference, body fat percentage, RDBP, HR, triglycerides, total cholesterol, 10-year cardiovascular risk, and heart/vascular age were found to be inversely related to lower CRF levels (all factors *p* < 0.0077). After adjusting for age and BMI, an inverse relationship between CRF and CVRF in police officers was obtained for HR and body fat (both factors *p* < 0.0116). In office workers, higher CRFs were significantly related to lower BMI, lower waist circumference, higher body fat, lower HR, lower triglycerides, lower LDL-cholesterol, and lower 10-year cardiovascular risk and heart/vascular age after adjusting the values for age (all factors *p* < 0.0314). Overall, a significantly lower waist circumference, lower body fat, lower total cholesterol, lower LDL-cholesterol, and lower 10-year cardiovascular risk and heart/vascular age were detected in office workers with higher CRF after adjusting for age and BMI (all factors *p* < 0.0405).

Based on the CRF and BMI, the participants of both groups (police officers and office workers) were divided into four level groups: low fitness (METs ≤ 12) and not obese (BMI < 30), fit (METs > 12) and not obese (BMI < 30), low fitness (METs ≤ 12) and obese (BMI ≥ 30), and fit (METs > 12) and obese (BMI ≥ 30). Only one police officer and two office workers were assigned to the group “fit and obese”. Consequently, no valid observations could be derived. Therefore, the group “fit and obese” was not considered in the corresponding Table 3, which presents the results of the four level groups and CVRF. We observed a large variance in the association between fitness level and body composition and CVRF in the groups of police officers and office workers.

In most cases, the police officers and office workers belonged to the “low fit and obese” group (police officers *n* = 33; office workers *n* = 25). “Low fitness and not obese” was the second largest group for both police officers (*n* = 12) and office workers (*n* = 13), followed by “fit and not obese” (police officers *n* = 9; office workers *n* = 6). A comparison of the four groups among police officers and office workers showed significant differences in the unadjusted model only for professional experience (police officers) and triglycerides (office workers) and only for RDBP (police officers) and professional experience (office workers) in the age-adjusted model. After adjusting for age and BMI, there was a significant difference for office workers only in total cholesterol. Participants showed significantly different parameters between the groups in body fat percentage, HR, triglycerides, total cholesterol, HDL-cholesterol, 10-year cardiovascular risk, and heart/vascular age (all of these parameters differed only for police officers) after consideration of the models with and without adjustment for age. Statistically significant differences in all three models were detected for the parameters of body fat percentage (police officers and office workers), HDL-cholesterol, and heart/vascular age (these parameters differed only in office workers). In all statistical tests, the parameters of RDBP for office workers and RSBP for both police officers and office workers were not different between the groups.

A linear regression model was developed to show the relationship between CVRF and CRF (MET) as continuous variables. These relationships for the group of police officers are presented in Table 4, while those for office workers are presented in Table 5.

Among police officers, significantly different relationships were observed between lower CVRF and CRF in the unadjusted model (*p* < 0.0199). Only the relationships between RSBP (*p* = 0.3823) and RDBP (*p* = 0.3089), and CRF in the unadjusted model were not significantly different. When adjusted for age, linear regression was not significantly different for the parameters of RSBP, RDBP, total cholesterol, LDL-cholesterol, and professional experience (*p* > 0.05). The other parameters showed a significant relationship between cardiovascular risk parameters and CRF (*p* < 0.0135). Linear regression after adjustments for age and BMI presented a significant relationship for the following parameters: body fat percentage, HR, 10-year cardiovascular risk, and heart/vascular age (all parameters *p* < 0.0218).

In the unadjusted model, office workers showed that every increase in METs was associated with a significant decrease in examined CVRF, except for RSBP, RDBP, and professional experience. A significant difference after adjustment for age was presented for CVRF BMI, waist circumference, body fat percentage, HR, HDL-cholesterol, LDL-cholesterol, and heart/vascular age (all parameters *p* < 0.0363). After adjustment for age and BMI, significant differences were detected for waist circumference, body fat percentage, RSBP, total cholesterol, HDL-cholesterol, and LDL-cholesterol in office workers (all parameters *p* < 0.0451), except for total cholesterol, LDL-cholesterol, and work experience. When adjusted for age and BMI, the values were only significantly lower for body fat percentage (*p* = 0.0061). The effects of fitness levels on waist circumference and body fat percentage were highly significant in the unadjusted and adjusted analysis for age (*p* <0.0001). In contrast, the effect was harmonized after an additional adjustment for BMI.

## 4. Discussion

The present study aimed to examine the influence of CRF on CVRF in different professions. We found that higher CRF is correlated with lower cholesterol levels and lower abdominal adiposity. One very important finding is that the 10-year cardiovascular risk (Framingham score) was lower in both groups. This shows that not only one’s profession but also one’s CRF is an important indicator for cardiovascular risk. Police officers and office workers who engage in sedentary work can improve their situation by training the cardiovascular system, e.g., in their leisure time.

Previous studies reported higher cardiovascular risk parameters among police officers and office workers with lower CRF [19,25]. The present study indicates that CRF has beneficial effects on CVRF for police officers and office workers. The study results showed that higher CRF levels in police officers and office workers (expressed as higher METs values) were significantly associated with lower values for BMI, waist circumference, body fat percentage, HR, total cholesterol, and 10-year cardiovascular risk as well as heart/vascular age (according to Framingham) after adjusting for age. Moreover, among police officers, we also verified the effects on the RDBP and triglyceride values. Several ongoing studies presented broad evidence for the positive effects of engaging in physical activity to reduce CVRF [26,27]. Another study on police officers in Switzerland also reported significantly lower cardiovascular risk scores and lower body fat percentages among police officers with higher CRF levels [28]. An especially significant reduction in RDBP in police officers was one notable observation because epidemiological studies demonstrated that even small blood pressure changes can have reducing effects on cardiovascular disease [29,30].

On the other hand, only 5.5% (*n* = 10) of police officers and 3.6% (*n* = 8) of office workers achieved METs > 12. The high rate of overweight and obese study participants may be responsible for the low number of high METs scores and the main reason for the low number of participants categorized under the “fit and not obese” group. The majority of police officers and office workers (police officers *n* = 33; office workers *n* = 25) were obese. High obesity rates in police officers have been reported in different countries by several studies [31,32]. Ramey et al. [33], moreover, demonstrated that police officers have higher obesity rates than the general public.

Research further suggests that a person’s focus on physical activity wanes over the course of his or her working life. In a Norwegian progress study, Lagestad et al. [34] examined police officers’ physical activity levels shortly before the end of their training period and after three years in police service. The authors showed that the frequency of physical activity and the importance of physical training decreased significantly over the course of the study. This study’s results are worrying, as the physical demands of police officers on duty are high [35].

Often, there are no fixed specifications for physical activity for police officers, so maintaining such activity is primarily contingent upon the intrinsic motivation of each individual. However, good CRF is necessary to perform well during emergency situations on patrol and to improve one’s own cardiovascular mortality risk. Corresponding studies by Lagestad et al. [34] and Bissett et al. [36] showed a positive attitude toward regular physical fitness check-ups. According to this study, police officers recognized the need for ongoing high fitness levels as an important criterion of their professional practice.

Apart from our results, the importance of physical activity for police officers has been underscored by the results of several studies. Steinhardt et al. [37] demonstrated that absenteeism among physically active police officers is lower than that among those who are seated, and Schilling et al. [38] emphasized the importance of high levels of CRF for the prevention of metabolic syndrome among police officers. This is an important factor, as the presence of a metabolic syndrome has been reported in up to 30% of police officers [39,40].

Regardless of this, it has long been known that physical inactivity is a changeable risk factor for cardiovascular diseases and many other chronic diseases, such as diabetes mellitus, obesity, high blood pressure, and various tumor diseases [41]. An unhealthy lifestyle and high health hazards were already reported among office workers [42,43], who seem to be at particularly risk for developing CVRF. Sedentary behavior thus seems to be an important factor for cardiovascular risk development in office workers [44,45]. For office workers, sedentism accounts for up to 81% of all work hours and is not generally compensated by physical activity during leisure time [46]. Katzmarzyk et al. [47] further reported a dose–effect relationship between sitting time and both mortality and the presence of cardiovascular risk.

In general, previous studies described a steady and pronounced decline in mean cardiorespiratory fitness in the general population over the past few years [48]. Previous studies described low CRF among various groups of office workers [8,10]. The importance of CRF for office workers was also shown in a study by Drake et al. [49], which presented significantly lower frequencies and durations of absence due to sickness among office workers with higher CRF.

The practical implications of our study are clear: Risk reduction in all professional groups is absolutely necessary. One possibility to reduce cardiovascular risk is to train CRF. We were able to achieve lower CVRF in those with higher CRF levels. In detail, every increase in CRF was associated with a significant decrease in important cardiovascular risk factors, especially a decrease in 10-year cardiovascular risk among office workers and police officers.

Physical activity programs are important implements to achieve positive effects for cardiovascular risk reduction. One approach is moderate-intensity physical activity, which was shown to yield significant clinical improvements in CRF among office workers [50]. Most notably, reductions in body weight, BMI, and fasting glucose levels were observed [51].

## 5. Strengths and Limitations

This study features some limitations that may impact the relevance of the results. This study was conducted with a small number of participants, and the examined groups of police officers and office workers presented only a small cohort of these two occupations. Therefore, extensive prospective studies with larger sample sizes are necessary to support or refute the results. Another limitation of this study is its lack of a description for work-associated physical activity in contrast to leisure-time physical activity.

However, the strengths of this study supersede its limitations. The most important strength is our observation that a significant reduction in cardiovascular risk can be accomplished if individual CRF is high, which can be achieved independent of the profession (e.g., police officers and office workers).

## 6. Conclusions

Both the police officers and office workers had a high rate of obesity and reduced CRF according to the assessment based on METs. This obesity led to increased cardiovascular risk. The study results mainly highlighted the beneficial effects of cardiorespiratory fitness in reducing cardiovascular risk and risk factors in both groups.

Improved CRF is needed for police officers and office workers because these professions entail sedentary work. The study results indicate that it would be of great importance to encourage movement among police officers and office workers to increase CRF for the purpose of reducing and preventing cardiovascular risk in those professions. One possible solution could be to establish worksite health-promoting initiatives, such as offering nutritional courses and exercise-promoting programs.

## Figures and Tables

**Table 1:** Baseline characteristics of the participants.

Police Officer (*n* = 55)			Office Worker (*n* = 46)	
Variables	Mean ± SD	Min–Max	Mean ± SD	Min–Max
Age	45.3 ± 7.8	25–58	45.8 ± 10.0	26–62
BMI (kg/m^2^)	28.0 ± 3.2	21.9–34.5	26.4 ± 4.1	21.1–43.0
Tobacco use (Cigarettes/day)	2.5 ± 6.0	0.0–20.0	1.7 ± 5.5	0.0–25.0
Body fat (%)	21.4 ± 5.6	8.2–34.8	20.8 ± 6.5	9.0–37.0
Waist circumference (cm)	97.8 ± 12.4	68–127	97.3 ± 11.7	80–132
Professional experience (years)	25.2 ± 8.4	5.0–41.0	21.1 ± 10.8	3.0–46.0
10-year cardiovascular risk (Framingham)	9.6 ± 7.4	1.2–30.0	9.7 ± 9.2	1.0–51.3
Heart/vascular age	51.1 ± 13.9	27–85	49.6 ± 15.5	26–86
METs absolute	9.7 ± 2.3	5.3–14.5	9.6 ± 2.3	5.4–13.8
Abs. VO2max (L/min.)	2.24 ± 0.73	0.82–4.65	2.04 ± 0.65	0.92–4.12
Rel. VO2max (mL/kg/KG)	34.1 ± 8.0	19.0–51.0	34.1 ± 8.1	19.0–49.0

**Table 2:** Cardiovascular risk factors of each MET category among police officers and office workers.

	METs ≤ 10	10 < METs ≤ 12	12 < METs ≤ 14	p1	p2	p3
	Police Officer *n* = 30,	Police Officer *n* = 15,	Police Officer *n* = 9,			
	Office Worker *n* = 27	Office Worker *n* = 11	Office Worker *n* = 8			
Age						
Police officer	48.6 (6.2)	44.3 (6.6)	36.9 (8.5)	0.0006	n/a	n/a
Office worker	47.4 (9.4)	42.9 (10.6)	44.6 (11.7)	0.4463	n/a	n/a
BMI, kg/m^2^						
Police officer	29.5 (3.2)	26.4 (2.4)	25.7 (1.5)	<0.0001	<0.0001	n/a
Office worker	27.9 (4.6)	24.3 (1.7)	24.3 (2.1)	0.0031	0.0032	n/a
Waist circumference, cm						
Police officer	104.3 (11.3)	91.2 (10.4)	87.5 (3.9)	<0.0001	0.0002	0.4296
Office worker	102.5 (11.6)	90.9 (6.5)	88.4 (7.6)	0.0002	0.0003	0.0355
Body fat, %						
Police officer	24.5 (4.2)	19.7 (4.3)	14.1 (3.8)	<0.0001	<0.0001	0.0020
Office worker	24.0 (6.1)	16.2 (2.1)	16.1 (5.5)	<0.0001	<0.0001	0.0031
RSBP, mmHg						
Police officer	129.3 (13.2)	126.0 (13.5)	123.9 (4.9)	0.1882	0.5133	0.8890
Office worker	130.0 (11.8)	129.1 (13.0)	126.2 (10.6)	0.6849	0.6895	0.4835
RDBP, mmHg						
Police officer	88.1 (11.7)	82.7 (8.8)	82.2 (4.4)	0.0748	0.0046	0.1055
Office worker	87.6 (8.5)	85.5 (9.3)	85.0 (9.3)	0.6795	0.7648	0.9294
HR, bpm						
Police officer	73.4 (11.6)	65.0 (6.8)	60.2 (10.9)	0.0022	0.0007	0.0116
Office worker	74.1 (13.7)	65.4 (6.8)	62.8 (8.6)	0.0118	0.0115	0.0581
Triglycerides, mg/dL						
Police officer	251.7 (166.8)	108.1 (42.9)	103.1 (43.8)	0.0002	0.0003	0.0655
Office worker	175.0 (90.7)	165.7 (103.1)	113.4 (67.5)	0.1101	0.1845	0.2274
Total cholesterol, mg/dL						
Police officer	220.9 (42.5)	194.5 (34.6)	175.8 (16.2)	0.0001	0.0077	0.0717
Office worker	213.4 (30.9)	203.5 (30.9)	188.0 (16.4)	0.0127	0.0087	0.0048
HDL-cholesterol, mg/dL						
Police officer	43.2 (11.9)	55.7 (15.5)	54.8 (11.8)	0.0052	0.0585	0.3814
Office worker	52.7 (12.2)	55.6 (18.7)	66.6 (14.0)	0.0431	0.0314	0.0715
LDL-cholesterol, mg/dL						
Police officer	142.1 (40.1)	120.5 (30.2)	103.8 (23.7)	0.0030	0.0844	0.3910
Office worker	125.7 (22.6)	114.9 (24.5)	100.0 (14.4)	0.0016	0.0033	0.0018
Professional experience, years						
Police officer	28.9 (6.4)	23.7 (8.0)	15.6 (7.6)	<0.0001	0.1472	0.2099
Office worker	20.7 (11.0)	21.0 (9.2)	22.6 (13.4)	0.9317	0.1771	0.5781
10-year cardiovascular risk (Framingham)						
Police officer	13.2 (8.0)	6.2 (3.6)	4.2 (3.0)	<0.0001	0.0031	0.0956
Office worker	11.8 (10.4)	7.7 (7.1)	5.3 (4.4)	0.0445	0.0128	0.0405
Heart/vascular age						
Police officer	58.3 (12.8)	45.3 (8.3)	38.6 (10.6)	<0.0001	0.0039	0.1750
Office worker	53.7 (15.2)	45.6 (16.0)	41.2 (12.1)	0.0483	0.0003	0.0023

p1: unadjusted; p2: adjusted for age; p3: adjusted for age and BMI.

**Table 3:** Participants (police officers and office workers) divided by METs and BMI in four different level groups (mean and SD).

	Low Fitness and Obese	Low Fitness and Not Obese	Fit and Not Obese	p1	p2	p3
	Police Officer *n* = 33,	Police Officer *n* = 12,	Police Officer *n* = 9,			
	Office Worker *n* = 25	Office Worker *n* = 13	Office Worker *n* = 6			
Age						
Police officer	48.4 (5.9)	44.0 (7.4)	36.7 (8.4)	0.0005	n/a	n/a
Office worker	47.9 (7.0)	42.6 (13.4)	44.3 (10.4)	0.3374	n/a	n/a
BMI, kg/m^2^						
Police officer	29.7 (2.8)	25.1 (1.7)	25.4 (1.3)	-	-	
Office worker	28.6 (4.4)	23.6 (1.1)	23.5 (1.7)	-	-	
Waist circumference, cm						
Police officer	105.3 (9.5)	85.1 (6.4)	87.2 (3.3)	-	-	-
Office worker	105.1 (9.8)	87.8 (2.8)	85.2 (5.1)	-	-	-
Body fat, %						
Police officer	24.7 (3.8)	18.2 (3.9)	15.3 (3.8)	<0.0001	<0.0001	0.0196
Office worker	24.3 (6.1)	16.8 (2.6)	14.3 (5.0)	<0.0001	<0.0001	0.0679
RSBP, mmHg						
Police officer	129.3 (12.8)	125.0 (14.5)	126.1 (7.0)	0.5021	0.6277	0.9738
Office worker	131.6 (12.8)	126.2 (9.6)	126.7 (12.1)	0.3185	0.2870	0.6495
RDBP, mmHg						
Police officer	87.7 (11.8)	82.5 (7.5)	83.3 (5.0)	0.1699	0.0228	0.5933
Office worker	89.0 (9.1)	83.1 (6.3)	86.7 (10.3)	0.0769	0.1205	0.7017
HR, bpm						
Police officer	73.1 (11.4)	63.9 (6.2)	62.0 (11.4)	0.0020	0.0010	0.0323
Office worker	73.8 (12.8)	67.5 (11.9)	62.3 (10.1)	0.0495	0.0326	0.1727
Triglycerides, mg/dL						
Police officer	238.1 (164.8)	109.5 (47.3)	95.4 (40.0)	0.0002	0.0015	0.8202
Office worker	196.0 (96.9)	126.8 (67.5)	120.7 (77.9)	0.0270	0.0960	0.0676
Total cholesterol, mg/dL						
Police officer	238.1 (164.8)	109.5 (47.3)	95.4 (40.0)	0.0002	0.0015	0.8202
Office worker	213.6 (29.8)	204.8 (33.1)	191.5 (17.1)	0.0589	0.0094	0.0109
HDL-cholesterol, mg/dL						
Police officer	43.8 (11.8)	56.9 (16.8)	60.2 (17.3)	0.0043	0.0493	0.7327
Office worker	48.8 (11.9)	62.8 (14.1)	64.5 (13.5)	0.0027	0.0029	0.0019
LDL-cholesterol, mg/dL						
Police officer	137.9 (39.7)	126.7 (33.4)	101.0 (22.7)	0.0024	0.1357	0.3920
Office worker	125.6 (23.3)	116.7 (23.4)	102.8 (15.8)	0.0193	0.0052	0.0040
Professional experience, years						
Police officer	28.2 (7.0)	24.3 (7.6)	15.3 (7.2)	0.0001	0.2303	0.2621
Office worker	20.3 (9.7)	21.7 (11.9)	23.7 (15.1)	0.8325	0.0284	0.2170
10-year cardiovascular risk (Framingham)						
Police officer	12.5 (8.0)	6.3 (3.7)	3.7 (2.9)	<0.0001	0.0305	0.5037
Office worker	12.5 (10.2)	7.1 (7.8)	5.0 (4.1)	0.0241	0.0120	0.0584
Heart/vascular age						
Police officer	57.1 (13.0)	45.6 (8.6)	36.8 (9.9)	<0.0001	0.0332	0.9770
Office worker	55.4 (13.7)	43.7 (16.9)	41.0 (10.6)	0.0102	0.0005	0.0066

p1: unadjusted; p2: adjusted for age; p3: adjusted for age and BMI.

**Table 4:** Linear regression models of cardiovascular risk factors and METs as continuous variable for police officers.

Police Officers	Model 1		Model 2		Model 3	
	Beta (SE)	*p*	Beta (SE)	*p*	Beta (SE)	*p*
Age	−1.81 (0.35)	<0.0001	n/a	n/a	n/a	n/a
BMI, kg/m^2^	−0.84 (0.15)	<0.0001	−0.94 (0.17)	<0.0001	n/a	n/a
Waist circumference, cm	−3.53 (0.61)	<0.0001	−3.38 (0.69)	<0.0001	−0.82 (0.46)	0.0797
Body fat, %	−1.74 (0.28)	<0.0001	−1.75 (0.31)	<0.0001	−0.92 (0.32)	0.0057
RSBP, mmHg	−0.67 (0.76)	0.3823	−0.69 (0.99)	0.4868	0.16 (1.07)	0.8846
RDBP, mmHg	−0.67 (0.65)	0.3089	−1.26 (0.71)	0.0792	−0.14 (0.89)	0.8756
HR, bpm	−2.68 (0.60)	<0.0001	−3.16 (0.66)	<0.0001	−3.33 (0.84)	0.0002
Triglycerides, mg/dL	−30.88 (8.95)	0.0011	−25.16 (8.59)	0.0050	−4.92 (6.94)	0.4816
Total cholesterol, mg/dL	−6.03 (2.05)	0.0047	−4.54 (2.43)	0.0673	−2.82 (2.85)	0.3269
HDL-cholesterol, mg/dL	3.24 (0.92)	0.0009	2.70 (1.06)	0.0135	1.36 (1.14)	0.2388
LDL-cholesterol, mg/dL	−5.36 (2.23)	0.0199	−3.15 (2.35)	0.1856	−0.93 (2.92)	0.7517
Professional experience, years	−1.95 (0.39)	<0.0001	−0.41 (0.30)	0.1700	−0.52 (0.41)	0.2121
10-year cardiovascular risk (Framingham)	−2.15 (0.39)	<0.0001	−1.30 (0.33)	0.0002	−1.10 (0.45)	0.0193
Heart/vascular age	−4.15 (0.66)	<0.0001	−2.22 (0.54)	0.0002	−1.65 (0.70)	0.0218

Model 1: linear regression, unadjusted; Model 2: linear regression, adjusted for age; Model 3: linear regression, adjusted for age and BMI.

**Table 5:** Linear regression models of cardiovascular risk factors and METs as continuous variables for office workers.

Office Workers	Model 1		Model 2		Model 3	
	Beta (SE)	*p*	Beta (SE)	*p*	Beta (SE)	*p*
Age	−1.07 (0.60)	0.0797	n/a	n/a	n/a	n/a
BMI, kg/m^2^	−1.03 (0.28)	0.0008	−1.02 (0.28)	0.0006	n/a	n/a
Waist circumference, cm	−3.55 (0.60)	<0.0001	−3.38 (0.58)	<0.0001	−1.26 (0.35)	0.0009
Body fat, %	−1.81 (0.35)	<0.0001	−1.87 (0.35)	<0.0001	−0.88 (0.29)	0.0039
RSBP, mmHg	−0.64 (0.93)	0.4932	−0.61 (0.90)	0.5001	1.46 (0.71)	0.0451
RDBP, mmHg	−0.53 (0.57)	0.3533	−0.43 (0.60)	0.4722	0.57 (0.66)	0.3879
HR, bpm	−1.79 (0.80)	0.0299	−1.91 (0.88)	0.0363	−1.62 (0.93)	0.0910
Triglycerides, mg/dL	−10.46 (4.86)	0.0370	−7.58 (5.08)	0.1428	−9.38 (6.40)	0.1500
Total cholesterol, mg/dL	−3.91 (1.65)	0.0221	−2.19 (1.43)	0.1328	−3.91 (1.71)	0.0276
HDL-cholesterol, mg/dL	2.39 (0.83)	0.0063	2.66 (0.86)	0.0035	3.00 (1.06)	0.0068
LDL-cholesterol, mg/dL	−4.08 (1.18)	0.0012	−3.16 (1.13)	0.0075	−4.79 (1.42)	0.0016
Professional experience, years	0.12 (0.67)	0.8623	0.96 (0.51)	0.0687	0.62 (0.69)	0.3726
10-year cardiovascular risk (Framingham)	−1.45 (0.69)	0.0400	−0.90 (0.58)	0.1260	−0.92 (0.80)	0.2514
Heart/vascular age	−2.66 (0.93)	0.0067	−1.36 (0.60)	0.0273	−1.20 (0.74)	0.1115

Model 1: linear regression, unadjusted; Model 2: linear regression, adjusted for age; Model 3: linear regression, adjusted for age and BMI.

## Data Availability

The datasets analyzed during the current study are available from the corresponding author upon reasonable request.

## Abbreviations

Abs.VO2max	Absolute maximal oxygen uptake
BMI	Body mass index
CRF	Cardiorespiratory fitness
CVRF	Cardiovascular risk factors
HDL-cholesterol	High-density lipoprotein cholesterol
HR	Heart rate
LDL-cholesterol	Low-density lipoprotein cholesterol
MET	Metabolic equivalent
OW	Office worker
PO	Police officer
RDBP	Diastolic resting blood pressure
Rel. VO2max	Relative maximal oxygen uptake
RSBP	Systolic resting blood pressure
